# The autoclaving and re-implantation of an infected prosthesis as a spacer during resection knee arthroplasty: a systematic review

**DOI:** 10.1007/s12306-021-00722-x

**Published:** 2021-07-28

**Authors:** Antonio Spinarelli, Davide Bizzoca, Lorenzo Moretti, Giovanni Vicenti, Raffaele Garofalo, Biagio Moretti

**Affiliations:** 1 Neuroscience and Sense Organs, Orthopaedic and Trauma UnitDepartment of Basic Medical SciencesSchool of Medicine, University of Bari Aldo Moro, AOU Consorziale “Policlinico”, Piazza Giulio Cesare 11, 700124 Bari, Italy; 2 Upper Limb Unit. “F Miulli” Hospital, Acquaviva Delle Fonti, Bari, Italy; 3 PhD Course in Public Health, Clinical Medicine, and Oncology, University of Bari “Aldo Moro, Piazza Giulio Cesare 11, 70100 Bari, Italy

**Keywords:** Periprosthetic joint infection, Total knee replacement, Two-stage revision strategy, Autoclaving, Re-use, Knee osteoarthritis

## Abstract

**Background:**

Hofmann et al., in 1995, first described an articulating spacer made by cleaning and autoclaving the original femoral component, which is then re-implanted with a new tibial polyethylene. This systematic review aims to assess the state of existing evidence on the intraoperative autoclaving and re-use of an infected prosthesis, as a spacer, during a two-stage revision following Periprosthetic Joint Infections (PJI).

**Methods:**

A systematic review was conducted with methods described in the Preferred Reporting Items for Systematic Reviews and Meta-Analyses. OVID-MEDLINE^®^, EMBASE, Cochrane Library, SCOPUS, Web of Science, Google Scholar and PubMed were searched from 1995 to April 2020 to identify relevant studies.

**Results:**

Fourteen studies were included in this systematic review: two prospective case series; six retrospective comparative studies and six retrospective case series. The reviewed studies included 567 patients (571 knees): 394 patients treated with autoclaved components and 173 with a spacer made of new components. The cumulative re-infection rate in patients treated with re-used autoclaved components was 13.7% (54 re-infections in 394 patients), whereas in control patients the re-infection rate was 13.3% (23 re-infections in 173 patients). The final Range of Movement in patients treated using the autoclaved components as a spacer, compared with patients receiving static spacers, was significantly higher in three out of four comparative studies.

**Conclusion:**

There is a moderate level of evidence that the intraoperative autoclaving and re-use of an infected prosthesis as a spacer, during a knee resection arthroplasty, is an effective procedure in the management of knee PJI.

## Introduction

Periprosthetic Joint Infection (PJI) is currently one of the most dreadful complications following total joint replacement (TJR) [1].

It is reported that PJI is the third most common cause of Total Hip Arthroplasty (THA) failure and the leading reason for Total Knee Arthroplasty (TKA) failure, accounting for 14.8% and 16.8% of all hip and knee revisions, respectively [1–3]. Nonetheless, a substantial increase in the prevalence of PJI is expected in the next years, mainly due to the increasing volume of TJRs performed all over the world, the emergence of resistant microorganisms and the tendency to perform joint arthroplasty even in patients with extensive comorbidities [4–6, 44, 45].

PJIs have a significant impact on the patient’s health status and quality of life, since they may cause severe pain, a progressive restriction of movement, feelings of isolation, depression, hopelessness and, if incorrectly managed, also a lethal epilogue [7, 8]. Thus, the Orthopedic community is paying great attention to the study and the treatment of this disease.

PJI may be classified, according to Zimmerli et al., into early, delayed and chronic infections [9]. Early PJIs occur within 3 months after TJR, whereas PJIs with onset between 3 and 24 months are classified as delayed infections and those occurring more than 24 months after TJR are classified as late [9].

In the management of chronic PJI, the two-stage revision strategy has evolved as the gold standard and preferred procedure, with a success rate exceeding 90% [10, 11]. In this procedure, the first step is the removal of infected prosthetic components and the concomitant implant of a cement spacer, followed by a period of tailored systemic antibiotic therapy [10, 11]. The re-implantation of revision components is then performed when the normalization of blood tests, synovial fluid analysis and local clinical signs of PJI are detected [10, 11]. Three meta-analyses have recently investigated the effectiveness of the two-stage surgical revision of the infected THA [8, 12] and TKA [13], compared with one-stage revision strategy, and both procedures resulted effective in the treatment of PJI in generally unselected patients [8, 12, 13].

Cement spacers are used in the first step of revision arthroplasty to maintain the joint space in distraction while providing high-dose local antibiotic delivery [11, 14]. They could be classified as static and articulating antibiotic-loaded spacers; it is reported that there is no significant difference between a non-articulating and an articulating spacer, in the treatment of PJI.

Static spacers, however, prevent joint movements until the second stage, and thus increase patient discomfort and may cause soft tissue contracture [15]. Therefore, articulating cement spacers have been developed to allow the patient to perform joint movements to some extent before the second-stage revision arthroplasty, therefore also preventing soft tissue contracture [16, 17].

Different types of articulating spacers have been described, including metal-on-polyethylene, cement-on-cement, or cement-on-polyethylene spacers [16, 17].

Hofmann et al. [18], in 1995, first described the treatment of an infected TKA using an articulating spacer made by cleaning and autoclaving the original femoral component. The autoclaved component was then re-implanted, with a new tibial polyethylene liner. These components are cemented into place using antibiotic-impregnated bone cement. To date, several studies have investigated the effectiveness of such a kind of spacer, but no randomized controlled trials have been conducted on these subjects.

This systematic review aims to assess the state of existing evidence on the intraoperative autoclaving and re-use of an infected prosthesis, as a spacer, during a knee resection arthroplasty performed for PJI.

## Methods

The study was conducted with methods described in the Preferred Reporting Items for Systematic Reviews and Meta-Analyses (PRISMA) [20, 21].

### Search strategy

OVID-MEDLINE®, EMBASE, Cochrane Library, SCOPUS, Web of Science, Google Scholar and PubMed were searched from 1995 to April 2020 to identify relevant studies for further analysis.

The main keywords were: “autoclaved” or “autoclaving” and “component” and “re-use”, or ‘periprosthetic joint infection’, or “total knee replacement”, or “total knee arthroplasty”, or “two-stage re-implantation”, or “articulating spacer”. A manual search of the reference lists of the selected publications was also performed, to identify additional studies for potential inclusion.

One review Author (BM) scanned the titles and abstracts. Potentially relevant articles were acquired for full-length text and Authors were contacted when the article was not available.

### Eligibility criteria

Full-text articles alone published between December 1995 and April 2020 were included. The review was restricted to articles published in English.

Inclusion criteria were: (1) all study designs; (2) detailed autoclaving procedure of the infected components; (3) sufficient data presented to estimate the re-infection rate and to assess the final clinical outcome.

Exclusion criteria were: (1) less than one year of follow-up, (2) re-use of components treated with other procedures than autoclaving and (3) lack of data about microorganism identification.

### Data extraction

Information was extracted from each study by one review Author (DB) and checked by another Author (AS), including: (1) characteristics of study participants (age, gender, duration of symptoms, microorganisms, follow-up) and the study inclusion and exclusion criteria; (2) autoclaving protocol of the infected components; (3) surgical therapy and antimicrobial treatment regimen; (4) treatment failure definition; (5) number of patients meeting the inclusion criteria; (6) outcomes and (7) re-infection rate. Disagreements were resolved by discussion between them.

### Study quality and risk of bias of the studies

The quality of each included study was assessed according to the AAOS clinical practice guideline and review methodology version 2, (available at www.orthoguides.org). The following points were evaluated: sample size and features; description of inclusion end exclusion criteria; blinding of participants and personnel (in randomized studies); appropriate statistical analysis; references of the study; data evaluation; the presence of bias; the presence of confounding factors; follow-up length.

Based on the depicted flaws and the study design, the quality of each study was defined as follows:

1. Prognostic study: high-quality study (< 1 flaw); moderate-quality study (≥ 1 and < 2 flaws); low-quality study (≥ 2 and < 3 flaws) and very low-quality study (≥ 3 flaws).
2. Diagnostic study: high-quality study (< 1 flaw); moderate-quality study (≥ 1 and < 2 flaws); low-quality study (≥ 2 and < 3 flaws) and very low-quality study (≥ 3 flaws).
3. Randomized study: high-quality study (< 2 flaws); moderate-quality study (≥ 2 and < 4 flaws); low-quality study (≥ 4 and < 6 flaws) and very low-quality study (≥ 6 flaws).
4. Observational study: high-quality study (< 2 flaws); moderate-quality study (≥ 2 and < 4 flaws); low-quality study (≥ 4 and < 6 flaws) and very low-quality study (≥ 6 flaws).

Two authors (L.M. and V. G.) independently evaluated all the studies. In case of disagreement between them, a new combined evaluation was performed.

The surgical procedures, the antibiotic regimen and the outcome definitions were evaluated in the included studies. Publication bias could not be assessed by a funnel plot considering the very low number of patients in each study.

### Primary outcome, secondary outcome

The primary outcome was to assess the re-infection rate in patients undergoing a two-stage revision strategy for PJI, using an articular spacer made by autoclaving the infected components. The second aim was to assess the final and intermediate functional outcomes, in patients undergoing this procedure.

## Summary measures

The cumulative re-infection rate was computed using extracted data from the relevant studies. It was defined as the number of re-infection during follow-up over the number of patients with chronic knee PJI treated with the two-stage revision strategy, using the autoclaved infected components as a spacer.

## Results

### Study selection

**Fig. 1:**
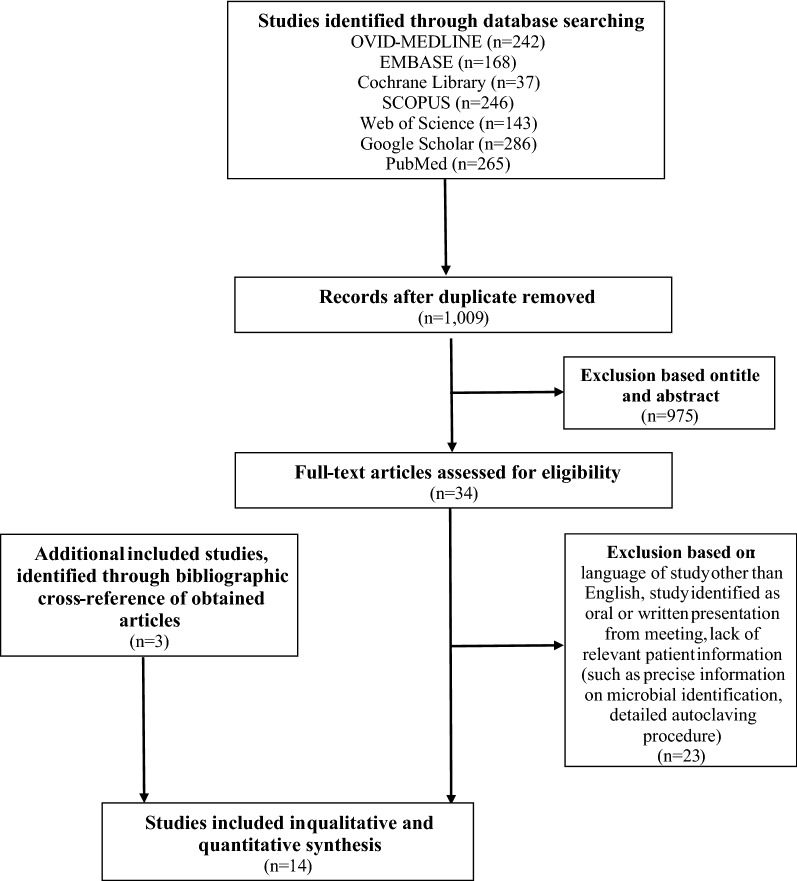
PRISMA flow diagram

The OVID-MEDLINE®, EMBASE, Cochrane Library, SCOPUS, Web of Science, Google Scholar and PubMed database searches provided a total of 1,387 studies for potential inclusion in the review (Fig. 1). After adjusting for duplicates, 1,009 studies remained. Of these, 975 studies were discarded after reading titles and reviewing abstracts. The Cochrane Library provided no relevant studies. Three additional abstracts were identified by checking the references of the relevant papers.

The full text of the remaining 34 studies was examined in greater detail. Of these, 23 studies did not meet the inclusion criteria. Moreover, three additional studies, identified through a bibliographic cross-reference of the obtained articles, met the inclusion criteria. Therefore, fourteen studies were finally included in this systematic review [14–16, 22–29, 33–35].

### Study quality

The process of quality assessment, performed according to the AAOS clinical practice guideline and review methodology version 2, depicted the following results: Four studies [15, 23–26] out of 14 (28.57%) were classified as moderate-quality studies, whereas 10 studies [14, 16, 22, 24, 27–29, 33–35] out of 14 (71.43%) were classified as high-quality studies (Table 2).

Due to the low number of patients included in each study, publication bias could not be assessed.

### Study characteristics

The study characteristics are summarized in Table-1 and Table-2. Twelve retrospective studies and two prospective case series were included. Five hundred and sixty-seven patients (571 knees) were included in this review. The number of patients, gender, age, mean follow-up, microorganisms identification, the time between first and second-step revision, outcomes at the follow-up and re-infection rates is reported in Table 1.

**Table 1:** Summary of the included clinical studies

Name of study	Design (level of evidence)	Years of inclusion	Number of knees (gender)	Age Mean (Range)	Control group	Follow-up Mean (Range)	Microorganisms identification	Time between the two stages	Outcomes at the final follow-up	Complications	Re-infection
Emerson et al. [15]	Retrospective comparative study (Level III)	1986–1999	48 knees (male: 17; female 31) Study group (autoclaved spacer): 26 Control Group (static spacer): 22	65.7	Static spacer	Study group: 3.8 years (2.6–6.4) Control Group 7.5 years (2.8–12.7)	42/48	Within 6 to 12 weeks	Study group: ROM: 107.8° Control Group ROM: 97.3°	Lethal cardiac complicatio*n* (*n* = 1)	Study group: 2/26 (7.7%) Control group 2/22 (9%)
Cuckler et al. [14]	Retrospective Case Series (Level IV)	1994–2002	44 Knees (Male: 13; Female: 31)	68 (44–92)	None	5.4 years (2–10)	38/44 (86.36%)	NA	ROM:120° (60°–130°) KS: 84 (45–98)	NA	1/44 (2.27%)
Hofmann et al. [22]	Retrospective Case Series (Level IV)	1989–2001	50 knees* (Male:25; Female:25)	67 (38–92)	None	73 months (24–150)	38/50 (76%)	NA	ROM: 4°–104° HSS:89 (70–100)	Revision for knee instability (*n* = 1) Knee arthrodesis (*n* = 1) Patellectomy for patella dislocation(*n* = 1) Vestibular damage because of vancomycin (*n* = 1)	6/50 (12%)
Huang et al. [23]	Retrospective Case Series (Level IV)	1996–2002	19 patients, 21 knees (Male: 5; Female 15)	68.7 (55–78)	None	52.2 (30–102)	15/21	20.7 weeks (6–92)	ROM: 97.6° (93°-120°) KS:80.6 (63–95) FS: 69 (50–90)	Lateral tilting of the patella (*n* = 3) Early radiolucent lines apparent beneath the medial tibial plate (*n* = 2)	1/21 (4.76%)
Jamsen et al. [24]	Retrospective comparative study (Level III)	1993–2003	32 Knees (Male:11; female:21) Study Group (Autoclaved Component): 22 Control Group (Cement Spacer):8	Study Group: 68 ± 10 (43–80) Control- Group: 70 ± 11 (43–85)	Cement Spacer	Study Group: 25 months (2–68) Control- Group: 48.9 months (2–86)	30/32 (93.75%)	Study Group: 170 ± 60 days (63–288) Control Group: 128 ± 56 days (69–223)	Study Group: ROM: 87.3° (55°-120°) KS:40.1 (10–73) FS:17.9 (0–60) Control Group: ROM: 44.3° (0°-95°) KS: 34 FS: 13 (0–45)	5 cases of spacer migration, associated with bone loss in one case	Study Group: 2/22 (9%) Control Group: 2/8 (25%)
Pietsch et al. [25]	Prospective Case Series (Level IV)	2000–2003	33 Knees (Male:16; Female:17)	65 (39–79)	None	28 months (12–48)	31/33 (93.9%)	15 weeks (7–28)	HSS:87 (53–97)	One case of temporary peroneal palsy One case of dislocation of the spacer due one fracture of the tibia	3/33 (9%)
Anderson et al. [26]	Retrospective Case Series (Level IV)	1997–2004	25 Knees (Male:10; Female: 15)	64 (45–87)	None	54 months (24–108)	20/25 (80%)	NA	ROM: 115° HSS: 91 (55–100)	NA	1/25 (4%)
Kalore et al. [27]	Retrospective comparative study (Level III)	2001–2009	53 knees (Male 38; Female 15) Study group (autoclaved component): 15 knees New femoral component and polyethylene insert (NFC) -Group: 16 knees Cement-on Cement (SMCs)-Group: 22 knees	64	NFC-Group: 16 knees SMC-Group: 22 knees	39 months Study Group: 73 months (37–105) NFC-Group: 19 months (12–32) SMC-Group: 32 months (14–56)	53/53 (100%)	Study Group:4.9 months NFC-Group: 2.7 months SMC-Group: 5.8 months	Study Group: MF. 95.7° NFC-Group: MF:98.3° SMC-Group: MF: 93.8°	Study Group: 2 cases of above-knee amputation NFC-Group: 1 case of spacer exchange and arthrodesis SMC-Group: 1 case of positive culture on knee aspiration	Study Group: 2/15 (13.3%) NFC-Group: 1/16 (6.25%) SMC-Group 2/22 (9%)
Kim et al. [28]	Retrospective Case Series (Level IV)	2006–2011	20 Knees (Male: 5; Female 15)	61.5 (36–75)	None	22.3 months (14–60)	16/20 (80%)	3.3 months (3–4)	ROM: 102.8° (80°-130°) HSS: 82.6 (56–100) KS:85.3 (77–94) FS:87.5 (70–100)	None	2/20 patients (10%)
Lee et al. [16]	Retrospective Case Series (Level IV)	2007–2011	19 Knees (Male: 3; Female: 16)	71 (63–75)	None	29 months (24–49)	9/20 (45%)	25 weeks (4–116)	ROM: 113° (90°–135°) HSS: 86 (56–100) KS: 82 FS: 54	Deep vein thrombosis (*n* = 2)	1/20 patients (5%)
Chen et al. [29]	Retrospective comparative study (Level III)	1999–2012 (Control group 199–2006; Study group 2006–2012)	18 Knees (Male:4 Female:14) Study Group: 10 patients treated with autoclaved spacer Control Group: 8 patients treated with static spacer	71.2 (20–88) Study Group:68.9 (20–88) Control Group:155.8 (49–420)	8 patients treated with static spacer (1999–2006)	Study Group: 32 months (24–46) Control Group: 40.8 months (25–56)	NA	Study Group: 135.9 days (61–296) Control group:155.8 (49–420)	Study Group: ROM: 94.5° (74–125°) KS:74.7 (62–88) Control Group: ROM: 74.3° (50°-90°) KS:71.4 (60–81)	Study Group: V–Y quadriceps plasties (*n* = 2); Wound dehiscence (*n* = 1) Control group: Deep Vein Thrombosis (*n* = 1)	Study Group: 2/10 (20%) Control Group: 1/8 (15%)
Nodzo et al. [33]	Retrospective comparative study (Level III)	2005–2014	140 knees (Male:90; Female: 50) Autoclaved group:39 patients (Male:20; female:19) Prefabricated spacer-group:58 patients (Male:43; female:15) Home-made mold-group: 43 patients (Male: 27; female: 16)	Autoclaved group: 67.8 ± 10.2 Prefabricated spacer-group: 65.3 ± 8.6 Home-made mold-group: 66.0 ± 11	Prefabricated space and Home-made mold	Autoclaved group: 52.4 ± 21.9 months Prefabricated space-group: 74.9 ± 35.1 months Home-made mold-group: 43.7 ± 16.7	NA	Autoclaved group: 11.6 w Prefabricated space-group: 10.7w Home-made mold-group: 10.0 w	Success rate: 87.2% in autoclaved-group; 89.7% in prefabricated-group; 95.3% in home-made mold-group	NA	Autoclaved group: 8/39 (20.5%) Prefabricated space-group: 10/58 (17.2%) Home-made mold-group: 5/43 (11.6%)
Goltz et al. [34]	Retrospective Case Series (Level IV)	2005–2015	59 knees (Male: 29; female:30)	61.0 ± 10.0 y–o-	None	5.0 ± 2.4 y	33 (67.35%)	5.6 ± 5.3 months	No re-infection: 37% (62.7)	Recurrence rate: 15%	22 (37.3%)
Kanas et al. [35]	Prospective case series (Level IV)	NA	10 knees (Male:6; female:4)	62.1 y.o	None	18.7 months	100%	6.5 months (only 3 remplanted; 7 kept the spacer)	9/10 out of infection (90%)	NA	Re-infection: 1/10 (10%)

*ROM* range of movement; *HSS* Hospital for Special Surgery knee score; *KS* Knee Society knee score; *FS* Knee Society Functional Score, *MF* mean flexion; *NA* not available

Table 2 shows the autoclaving protocol, articular spacer details (the type of femoral component, type of tibial insert and antibiotic-impregnated cement features) and the antibiotic treatment performed. In all the studies, the patients underwent a two-stage revision strategy for knee PJI, using a spacer made by autoclaving the infected components. In six studies out of fourteen (42.86%), the antibiotic-impregnated articular spacer was realized using an autoclaved femoral component and a new tibial polyethylene insert [15–22, 26–28, 34, 35]; in four studies out of fourteen (28.57%), both the femoral component and the tibial insert were autoclaved and re-used [14, 23–25] and in four studies out of fourteen (28.57%), a metal-on-cement spacer was implanted [16, 27, 29, 33].

**Table 2:** Details of the included clinical studies

Name of study	Autoclaving protocol	Type of femoral component	Type of tibial insert	Antibiotic-impregnated cement features	Antibiotic therapy	Study quality
Emerson et al. [15]	Autoclaving of the femoral component, with an undetailed protocol	Metal-on-polyethylene cemented spacer	New tibial insert and patella	The cement contained 3.6 g. tobramycin and 2 g vancomycin per 40 g. package for each spacer technique	Tailored intravenous antibiotic therapy	Moderate-quality study
Cuckler et al. [14]	Autoclaving of the femoral component and tibial polyethylene for 10 min	Metal-on-polyethylene cemented spacer	Autoclaved tibial polyethylene insert	There were 4.8 g. of tobramycin in each 40 g. of bone cement	For 6 weeks	High-quality study
Hofmann et al. [22]	Autoclaving of the femoral component with an undetailed protocol	Metal-on-polyethylene cemented spacer	New tibial polyethylene insert and sometimes (40%) a new polyethylene patella component	Antibiotic-impregnated cement: with tobramycin in a ratio of 4.8 g. tobramycin to 40 g. cement	Intravenous antibiotics specific to the organism	High-quality study
Huang et al. [23]	Autoclaving of both femoral component and polyethylene insert with an undetailed protocol	Metal-on-polyethylene cemented spacer	Autoclaved polyethylene insert	The cement contained 1 g. of tobramycin per 40-g package of cement	For 6 weeks	Moderate-quality study
Jamsen et al. [24]	Autoclaving of both femoral component and polyethylene insert with an undetailed protocol	Metal-on-polyethylene cemented spacer	Autoclaved polyethylene insert	Antibiotic-impregnated cement: 2–4 g. antibiotics per 40 g. of cement powder	Minimum for 5 weeks, with at least 2 weeks of parenteral antibiotics	High-quality study
Pietsch et al. [25]	Autoclaving of both femoral component and polyethylene insert with an undetailed protocol	Metal-on-polyethylene cemented spacer	Autoclaved polyethylene insert	Antibiotic-impregnated cement: 2–4 g. antibiotics per 40 g. of cement powder	The peri-/post-operative systemic antibiotic regimen is decided concerning the antibiotic spectrum	Moderate-quality study
Anderson et al. [26]	NA	Metal-on-polyethylene cemented spacer	A new polyethylene insert	Antibiotic-impregnated cement: 3.6 g. tobramycin and 2 g. vancomycin per 40 g. the package was used in 22 cases. Three cases had tailored antibiotics	Appropriate antibiotics were administered intravenously for six weeks postoperatively	Moderate-quality study
Kalore et al. [27]	The femoral component was scrubbed with betadine, then autoclaved After 2006 in this institution was prohibited the re-use of explanted components	Metal-on-cement spacer	-	Four packs of bone cement with four 1.0-g. doses of tobramycin powder and four 1.5-g. doses of vancomycin powder	At least 6 weeks of appropriate intravenous antibiotic therapy	High-quality study
Kim et al. [28]	Autoclaving of the femoral component at 137 °C for 7 min	Metal-on-polyethylene cemented spacer	A new polyethylene insert	4 g. Vancomycin + 1 g. gentamycin; otherwise, a mixture of cephalosporin antibiotics sensitive to the cultured organism was used	Antibiotic therapy for 10 weeks: intravenously for 6 weeks and orally for the following 4 weeks	High-quality study
Lee et al. [16]	Autoclaving of the femoral component at 132 °C for 30 min	Metal-on-cement spacer	-	4 g. Vancomycin + 4 g. 1^st^ generation cephalosporin + 0.5 g. gentamicin; appropriate antibiotic was added if causative bacteria had been identified previously	Suitable antibiotics (or empiric antibiotics if no causative bacterium was identified) for 4–6 weeks by intravenous injections	High-quality study
Chen et al. [29]	Study Group: Autoclaving of the femoral component at 137 °C for 7 min	Study Group: Metal-on-cement spacer Control group: Static Spacer	-	Antibiotic-impregnated cement	Suitable antibiotics administered for 2 weeks intravenously, then for 4 weeks orally	High-quality study
Nodzo et al. [33]	NA	Autoclaved femoral component	Antibiotic-loaded cement	Tobramycin-impregnated cement	Suitable antibiotics administered for 6 weeks	High-quality study
Goltz et al. [34]	NA	Autoclaved femoral component	A new polyethylene insert	Dosing varied between 1–3 g vancomycin, 160–240 mg liquid gentamicin, and/or 2.4–3.6 g tobramycin with each 40 g cement package	All patients underwent at least 6 weeks of parenteral antibiotics, specialist followed by an antibiotic holiday of 3–4 weeks	High-quality study
Kanas et al. [35]	The explanted femoral and tibial components were cleaned manually s to remove any visible adherent bone and cement. The components were then scrubbed with a chlorhexidine brush to remove any visible soft tissue. The components were then placed in the operating room autoclave and flash-sterilized on a standard gravity-displacement cycle at 15 min (132 °C, 32PSIG)	Autoclaved femoral component (Autoclaved tibial component sent for sonication)	New tibial component	Bone cement was mixed with 2 g of vancomycin powder and 160 mg of liquid gentamicin per bag	The patient received a minimum of 6 weeks of intravenous antibiotics per infectious disease recommendations based on the isolated organism	High-quality study

All the patients received an antibiotic-impregnated cement in the spacer, as well as adequate antibiotic therapy for at least five to six weeks (Table-2) [14–16, 22–29]. The autoclaving protocol was specified only in four studies out of fourteen (28.57%) [16, 28, 29, 35]. Two studies out of fourteen (14.28%) indicated only the duration time of the autoclaving process [14, 35], whereas it was not detailed in the remaining studies [22–27, 33, 34]. In all the studies, the implant was mechanically cleaned of all cement and tissue before undergoing autoclaving.

### The cumulative rate of re-infection

The reviewed studies included 567 patients (571 knees): Three hundred and ninty-four patients treated with autoclaved components and 173 with a spacer made by new components. The cumulative re-infection rate in patients treated with re-used autoclaved components was 13.7% (54 re-infections in 394 patients), whereas in control patients, the re-infection rate was 13.3% (23 re-infections in 173 patients).

### Mobile versus static articular spacers

Patients treated with mobile articular spacers, made by autoclaving the infected components, showed, after the spacer implantation, a significant higher ROM, compared with those treated with static spacers, in one out of four comparative studies reviewed [27].

The final ROM in patients treated using the autoclaved components as a spacer, compared with subjects receiving static spacers, was significantly higher in three out of four comparative studies [15, 24, 29]. However, at final follow-up, the functional scores -i.e., Hospital for Special Surgery Knee Score (HSS), Knee Society Knee Score (KS) and Knee Society Functional Score (FS)-registered in patients treated with articular spacers, compared with static spacers group, showed no significant difference in all the reviewed comparative studies.

## Discussion

### Summary of evidence

PJI currently represents the leading cause of TKA failure and a further increase of prevalence is expected in future years [40–45]. Consequently, the management of an infected prosthesis is a hot topic in orthopedics.

Hofmann et al. [18], in 1995, first described the treatment of an infected TKA using an articulating spacer made by cleaning and autoclaving the original femoral component. This systematic review aims to assess the state of existing evidence on the intraoperative autoclaving and re-use of an infected prosthesis, as a spacer, during a knee resection arthroplasty performed for PJI.

The review results suggest that the intraoperative autoclaving and re-use of an infected prosthesis as a spacer, during a knee resection arthroplasty performed for PJI, is an effective strategy. Hence, a comparable re-infection rate was observed in patients managed with autoclaved components compared with patients treated with a new spacer. Moreover, patients receiving mobile articular spacers showed a better functional outcome at the final follow-up.

In this procedure, before undergoing autoclaving, the infected femoral component should be mechanically cleaned of all cement and tissue [30, 37–39]. The autoclave should be near the operating room to facilitate aseptic delivery to the sterile field; the use of a rigid, re-usable sterilization container system is recommended [30]. If a spore test (it takes approximately one hour after the cycle) is not able to be run before implant use, then the implant should receive the equivalent of full-cycle steam sterilization and not a flash sterilization cycle [30].

Patients treated using this procedure showed a comparable re-infection rate to those undergoing a two-stage revision strategy, using a sterile cement spacer. The cumulative re-infection rate, at a minimum of two years follow-up, was 13.7% in patients treated with an autoclaved prosthesis and 13.3% in patients receiving a sterile cement spacer.

Pietsch et al. [25], in a prospective non-randomized trial on 33 patients with knee PJI undergoing two-stage revision using autoclaved both femoral component and polyethylene tibial insert, reported an infection rate of 9% (3 re-infections out of 33) at a mean 28-month follow-up.

Kanas et al. [35] have recently performed a prospective case series, recruiting 10 patients with knee PJI. All the patients were managed with TKA explantation, debridement, and placement of an articulating antibiotic spacer consisting of the explanted and sterilized femoral component and a new polyethylene tibial insert [35]. Only 3 patients out of 10 were re-implanted, while the reaming 7 patients kept the spacer. At the final follow-up, a re-infection rate of 10% was observed [35].

In the retrospective comparative studies, the re-reinfection rates resulted not significantly different in patients treated with an autoclaved component with respect to those receiving new sterile spacers.

Emerson et al. [15], in a retrospective study comparing 26 patients treated with autoclaved components to 22 with sterile static spacers, reported a re-infection rate of 7.7% in the autoclaved components group (2 patients out of 26), at 3.8 years mean follow-up, and a re-infection rate of 9% (2 patients out of 22), at 7.5 years mean follow-up, in patients receiving sterile spacers. The Authors specified that there was not the same organism, in the re-infected patients treated with mobile spacers made by autoclaving the infected components [15].

Jamsen et al. [24] observed a re-infection rate of 9% (2 patients out of 22), at 25 months mean follow-up, in patients treated with re-sterilized prosthesis components and a re-infection rate of 25% (2 patients out of 8), at 48.9 months mean follow-up, in patients treated with cement spacers.

Kalore et al. [27] showed a 13.3% re-infection rate (2 patients out of 15), at mean 73-month follow-up, in patients operated on with autoclaved femoral component, a re-infection rate of 6.25% (1 patient out of 16), at a mean 19-month follow-up, in the group treated with a new femoral component and a 9% re-infection rate (2 patients out of 22), at mean 32-month follow-up, in subjects treated with silicone mold component spacers.

Chen et al. [29] reported a re-infection rate of 20% (2 patients out of 10), at 32-month mean follow-up, in patients treated with autoclaved femoral component and tibial insert and a re-infection rate of 15% (one patient out of 8), at 40.8 months mean follow-up, in those with sterile static spacers.

Nodzo et al. 2017 [33], in a retrospective comparative study including 140 patients with knee PJI, divided into three groups (i.e., autoclaved-group: 39 patients; prefabricated spacer-group:58 patients and home-made mold spacer-group: 43 patients), observed no statistically significant difference in the success rates between groups. A re-infection rate of 20.5% was observed in the autoclaved-group at the final follow-up.

This procedure has a good cost-effectiveness ratio, since it is reported that a spacer made by autoclaving the infected components has a direct cost of $932, whereas spacers made by new femoral component cost $3589 and molded cement spacers cost $3945 [27]. It is also reported that the temporarily re-use of the femoral component can reduce the cost of the articulating spacer by approximately $1900/case, versus a new femoral component, and by approximately $1000/case, versus a molded cement spacer [30]. Consequently, this technique is a safe and cost-effective option to improve patient function during revision for PJI.

The value of these recommendations has been also confirmed by the data deriving from an in vitro and in vivo study [30]. Lyons et al. recently showed that six cobalt-chrome femurs components, explanted from patients with knee PJI, became sterile after autoclaving under a standard gravity-displacement cycle (132 °C, 27 PSIG, 10 min) [30].

Moreover, these Authors conducted an in vitro test on six sterile chrome cobalt femur implants (2 cementless, 4 cemented), inoculated with different bacterial species. After that, three of these components were autoclaved on a standard gravity–displacement vacuum cycle (121 °C, 15 PSIG, 45 min), while the remaining implants were maintained in a sterile environment at room temperature. All these components were subjected to 5 min of sonication; the diluted sonicate of the autoclaved components showed no bacterial growth on an agar plate, whereas the control components, that did not undergo autoclaving treatment after inoculation, highlighted growth of multiple colonies of the original bacteria [30]. Finally, the biofilm in vitro test of three MRSA biofilm covered cobalt-chrome pieces that underwent autoclave treatment, showed a statistically significant reduction of relative biofilm compared to controls [30]. The biofilm burden reduction was also confirmed by Scanning Electron Microscope images [30].

Interestingly, Park et al. [36] have evaluated the role of sonication in depicting the sterility of an autoclaved femoral component explanted from an infected TKA. These authors found only two infected femoral components out of nineteen (10.53%) after sonification [36]. Hence, they concluded autoclaving of an infected femoral implant could be a good method for using the temporary articulating antibiotic spacer in two-stage revision arthroplasty [36].

Similar positive findings have been reported by Nodzo et al., in a prospective observational study [33]. These authors cleaned and autoclaved both the explanted femoral and tibial components. Then, the autoclaved femoral components were re-implanted, while the tibial ones were aseptically packaged and sent to a microbiology lab for sonication and culture of the sonicate for 14 days; all the cleaned tibial components were negative for bacterial growth of the infecting organism after final testing and analysis [33].

Unfortunately, the relatively low number of patients in our review, as well as the absence of randomized controlled trials among the reviewed articles, allow us to recommend a moderate level of evidence. Furthermore, it should be remarked that the Center for Disease Control (CDC), Association of Operating Room Nurses (AORN), health care institutions, implant companies and medical consult teams are hesitant to temporarily re-use implants for medical, legal and financial reasons [31, 32].

Moreover, other relevant concerns should be considered when performing this procedure, including a lack of guarantee of the re-used component sonication, the potential delayed surgical time because of the explanted component autoclaving and the off-label implant use, that might raise potential medicolegal issues.

It should be noted, however, that all the reviewed studies showed the re-use of the autoclaved component as a spacer is an effective procedure in the eradication of knee PJI.

### Strengths and limitations

To our knowledge, this is the first systematic review, which aims to assess the role of intraoperative autoclaving and re-use of an infected prosthesis as a spacer during knee resection arthroplasty. However, its limitations need to be considered.

- First, although fourteen studies were included in this review, no controlled trials were identified.
- Most studies were retrospective case series; therefore, they were level IV studies.
- The reviewed studies have a different length of follow-up.
- The included studies have a low number of patients. The patients’ characteristics, the autoclaving procedure the cement spacer features differed across the reviewed studies.
- The antibiotic treatment performed after the prosthesis removal was not standardized among the studies; this feature could significantly influence the outcomes of a two-stage revision strategy.
- Several papers do not detail the adopted autoclaving protocol; it is impossible to assess if a different autoclaving protocol could influence the re-infection rate. Future studies are needed to define a standardized autoclaving protocol.

## Conclusion

The intraoperative autoclaving and re-use of a removed infected prosthesis, as a spacer, during a knee resection arthroplasty performed for PJI is an effective procedure in the management of knee PJI. This procedure has a reported re-infection rate ranging from 2.27 to 37% and a cumulative re-infection rate of 13.7% [14–16, 22–29, 33–35]; no significant differences between patients treated with autoclaved components and those with sterile static spacers were founded in the reviewed comparative studies.

The final ROM in patients treated using the autoclaved components as a spacer, compared with patients receiving static spacers, was significantly higher in three out of four comparative studies.

However, no prospective randomized controlled trials have focused on this subject; therefore, the data showed in this review have a moderate level of evidence.
